# Early detection of cardiovascular disease in chest population screening: challenges for a rapidly emerging cardiac CT application

**DOI:** 10.1093/bjr/tqaf195

**Published:** 2025-08-18

**Authors:** Anna N H Walstra, Jan Willem C Gratama, Marjolein A Heuvelmans, Matthijs Oudkerk

**Affiliations:** Department of Public Health, Erasmus Medical Center Rotterdam, Rotterdam 3015 GD, The Netherlands; Research Institute for Diagnostic Accuracy, Groningen 9713 GH, The Netherlands; Research Institute for Diagnostic Accuracy, Groningen 9713 GH, The Netherlands; Department of Radiology and Nuclear Medicine, Gelre Ziekenhuizen, Apeldoorn 7334 DZ, The Netherlands; Research Institute for Diagnostic Accuracy, Groningen 9713 GH, The Netherlands; Department of Epidemiology, University of Groningen, University Medical Center Groningen, Groningen 9713 GZ, The Netherlands; Department of Respiratory Medicine, Amsterdam University Medical Center, Amsterdam 1105 AZ, The Netherlands; Research Institute for Diagnostic Accuracy, Groningen 9713 GH, The Netherlands; Faculty of Medical Sciences, University of Groningen, University Medical Center Groningen, Groningen 9713 GZ, The Netherlands

**Keywords:** screening, cardiovascular disease, lung cancer screening, CT, cardiac CT, coronary artery calcium

## Abstract

While lung cancer screening (LCS) reduces lung cancer-related mortality in high-risk individuals, cardiovascular disease (CVD) remains a leading cause of death due to shared risk factors such as smoking and age. Coronary artery calcium (CAC) assessment offers an opportunity for concurrent cardiovascular screening, with higher CAC scores indicating increased CVD risk and mortality. Despite guidelines recommending CAC-scoring on all non-contrast chest CT scans, a lack of standardization leads to underreporting and missed opportunities for preventive care. Routine CAC-scoring in LCS can enable personalized CVD management and reduce unnecessary treatments. However, challenges persist in achieving adequate diagnostic quality with one combined image acquisition for both lung and cardiovascular assessment. Advancements in CT technology have improved CAC quantification on low-dose CT scans. Electron-beam tomography, valued for superior temporal resolution, was replaced by multidetector CT for better spatial resolution and general usability. Dual-source CT further improved temporal resolution and reduced motion artifacts, making non-gated CT protocols for CAC-assessment possible. Additionally, artificial intelligence-based CAC quantification can reduce the added workload of cardiovascular screening within LCS programs. This review explores recent advancements in cardiac CT technologies that address prior challenges in opportunistic CVD screening and considers key factors for integrating CVD screening into LCS programs, aiming for high-quality standardization in CAC reporting.

## Introduction

Lung cancer is the second most common malignancy and remains the leading cause of cancer-related deaths worldwide.^1^ Large-scale lung cancer screening (LCS) trials, such as the National Lung Screening Trial (NLST) and the Dutch-Belgian Randomized Lung Cancer Screening Trial (NELSON), have demonstrated the effectiveness of low-dose CT (LDCT) in LCS, significantly reducing lung cancer-related mortality among high-risk individuals.^2^^,^^3^ However, cardiovascular disease (CVD) remains the leading cause of mortality worldwide and was a major contributor to deaths among participants in the NLST-LCS cohort.^3^^,^^4^ Given the shared risk factors—such as smoking history and increasing age—LCS populations are at a disproportionately high risk for CVD-related morbidity and mortality.^5^

This overlap presents a crucial opportunity to integrate cardiovascular screening into LCS programs, potentially reducing CVD mortality. Since the heart is inherently visualized on chest CTs, cardiovascular risk assessment can be included without additional imaging. The coronary artery calcium (CAC) score correlates strongly with plaque burden and is a well-established predictor of CVD risk.^6^^,^^7^ For instance, a CAC score of 1000 is associated with a 10-fold increased risk of all-cause mortality, while a score of 0 indicates low risk, comparable to that of the general population.^8^ Thus, incorporating CAC screening into LCS programs allows the identification of low- and high-risk individuals, facilitating personalized primary prevention for CVD in LCS participants.^9^

Coronary calcium was initially measured using electron beam tomography (EBT), which was the standard in cardiac imaging, and later with multidetector CT (MDCT), both employing prospective electrocardiographic (ECG) triggering to reduce cardiac motion artifacts.^10–13^ However, CAC scoring could not be integrated with non-gated LDCT due to limited temporal resolution, which resulted in significant motion artifacts. Recent advancements in CT imaging technology, such as wide-detector configurations, dual-source CT (DSCT), and photon-counting CT systems, have significantly improved temporal resolution and expanded the capabilities of cardiac imaging. These innovations provide an opportunity for accurate CAC-scoring on non-ECG-gated, low-dose chest CT scans, providing a viable solution for opportunistic CVD screening within LCS programs.^14–19^

Integrating CAC quantification into LCS programs demands accurate and reliable cardiovascular risk assessments using 1-run, non-ECG-gated, low-dose chest CT. This review discusses advancements in cardiac CT technologies that address prior challenges for opportunistic cardiovascular screening, along with key considerations for the integration of cardiovascular screening within LCS programs.

## CAC as biomarker

CAC is a reliable marker of atherosclerosis, reflecting the buildup of calcified plaque in the coronary arteries.^7^ Two primary methods are used for CAC scoring: automatic quantification (Agatston score, volume score, and mass score) and visual assessment.^20–23^ The Agatston score is the most widely used method and serves as the reference for population databases and cardiovascular risk stratification due to its scientifically proven strong predictive value for cardiovascular and all-cause mortality.^6^^,^^9^^,^^22^^,^^24^ The latter 2, volume score and mass score, have been less studied than the Agatston score with regard to their predictive value. However, the Agatston score is more variable than the volume and mass scores, as it depends on a weighting factor based on calcium density, which can vary between CT protocols.^16^^,^^18^^,^^25^^,^^26^ While automatic quantification methods are readily accessible, they require specialized software for computation. The visual assessment method, which classifies CAC as none, mild, moderate, or severe, offers the quickest evaluation and is preferred when automatic software is unavailable.^20^^,^^27^^,^^28^ Despite its simplicity, visual assessment correlates well with both Agatston scores and mortality outcomes using either dedicated cardiac CT or chest LDCT scans. It serves as a practical alternative in resource-limited settings, though its accuracy can vary with the reader’s experience.^29–31^

CAC is as an independent risk factor for CVD, even in asymptomatic individuals, with higher scores indicating a greater risk of cardiac events.^8^^,^^32^ Moreover, CAC burden increases exponentially with age, with higher scores signifying a progressively greater risk of cardiovascular mortality.^33^ While any detectable CAC strongly correlates with increased CVD risk, studies have shown that individuals with a CAC score of 0 are at very low risk for CVD, regardless of age.^33–35^ CAC quantification allows for the classification of asymptomatic patients into low-, intermediate-, and high-risk categories. In 2018, the American College of Cardiology/American Heart Association Task Force updated the Guidelines on Management of Blood Cholesterol to recommend CAC as a valuable tool for determining statin use, advising statin therapy for any individual with an Agatston CAC score of ≥100, unless otherwise deferred by the outcome of clinician-patient risk discussion.^36^ CAC plays a crucial role in guiding personalized cardiovascular care, providing clinicians the tools to alter statin therapy via personalized CAC scores and associated risk category.^37^ Preliminary results from the European LCS trial 4-IN-THE-LUNG-RUN (4ITLR) show CAC is present in 85% of individuals, of which half are candidates for preventive CVD treatment. About 15% had a CAC score of 0, exempting them from CVD preventive medication as per existing guidelines.^38^ Multiple studies have demonstrated that reporting CAC in LCS led to changes in the management of participants with previously undiagnosed CVD, primarily leading to increased prescription of statin.^39^^,^^40^ A recent study from the Ontario Health Lung Cancer Screening Pilot found that CAC was present in 82.9% of participants. Extensive CAC (estimated Agatston score >400, present in 29.5% of participants) was strongly associated with all-cause mortality and cardiovascular events, remaining a significant predictor even after adjusting for non-cardiovascular causes of death.^41^ Also within the NELSON LCS trial, CAC was found to be a strong predictor of cardiovascular events, even beyond age, pack-years, and smoking status.^42^ In the ITALUNG LCS trial, reduced cardiovascular mortality in the LDCT group may be linked to reporting the presence of CAC, prompting preventive interventions.^43^ Furthermore, Mitchell et al^44^ demonstrated that statin therapy effectively reduced the risk of major adverse cardiovascular events (MACEs) in individuals with CAC, with the greatest benefit observed in those with a CAC score >100. In contrast, individuals without detectable CAC showed no reduction in MACEs from statin therapy. While a CAC score of 0 suggests a low likelihood of significant CVD, non-calcified atherosclerosis may still be present, underscoring the importance of a comprehensive risk assessment.^45^ Evaluation of CAC progression through follow-up measurements can provide insight into changes in CVD risk. CAC progression has been linked to a worse prognosis, as studies have demonstrated its value over baseline CAC in predicting all-cause mortality and cardiovascular events.^46^^,^^47^ Several methods for assessing CAC progression remain under debate, including absolute change, percentage change, or combined approaches, as alternatives to shifts in Agatston risk categories.^48^ These more graduated methods may be preferable, particularly in borderline cases that are prone to measurement variability. However, others showed that if serial CAC scanning is performed, the latest scan should be used for risk assessment, and CAC progression provides minimal additional prognostic information.^49^^,^^50^ In addition, CAC progression may also indicate plaque stabilization, particularly following statin therapy.^51^ The overall evidence remains inconclusive, and the clinical significance of CAC progression has yet to be fully established.^52^^,^^53^

The Framingham risk score (FRS) is a widely used model that estimates 10-year coronary event risk in individuals without prior CVD. It incorporates variables such as age, sex, smoking history, blood pressure, cholesterol levels, high-density lipoprotein cholesterol, and blood glucose levels or diabetes history. Integrating CAC score with FRS improved CVD risk prediction in higher-risk categories (FRS ≥10%) but was less impactful in low-risk categories (FRS <10%).^54^ CAC-scoring also improved risk prediction in the elderly, where a high calcium score (>1000) implied a high risk of cardiovascular events, regardless of the FRS.^55^ Thus, beyond independently predicting CVD risk, CAC scoring is a valuable addition to traditional risk models.^54^^,^^55^ In Europe, the Systematic Coronary Risk Evaluation (SCORE) chart is the most commonly used risk model, similar to FRS, estimating fatal CVD risk within 4 years.

The 2014 Risk or Benefit in Screening for Cardiovascular Disease (ROBINSCA) trial randomized approximately 39 000 participants into 3 groups: 1 screened for cardiovascular risk using the SCORE chart, another using CAC scoring via CT, and a control group with no screening. They received preventive treatment according to their risk group.^56^ CAC scoring classified fewer participants as high risk and reduced the number of individuals recommended for preventive treatment compared to SCORE. Mortality and MACE data have not been published yet but will reveal whether CAC is a robust risk predictor.^57^ Similarly, recent research showed that adding CAC scoring to a risk factor-weighted clinical likelihood (RF-CL) model improved risk stratification, particularly by identifying more low- and moderate-risk patients unlikely to benefit from further testing, while also demonstrating a stronger correlation with obstructive coronary artery disease prevalence than RF-CL alone.^58^

## Current guidelines and recommendations

In recent years, multiple recommendations emphasized the importance of reporting CAC scores on all chest CT scans where feasible. The consensus statement of the British Society of Cardiovascular Imaging/British Society of Cardiac Computed Tomography and British Society of Thoracic Imaging recommend reviewing the heart in all CT scans when included in the field-of-view.^59^ Similarly, the American College of Radiology (ACR) expects radiologists to report CAC using a simple visual quantification scale (none, mild, moderate, severe) on all non-contrast CT scans.^60^ The 2016 Society of Cardiovascular Computed Tomography (SCCT) and the Society of Thoracic Radiology (STR) guidelines also advise reporting CAC on all non-contrast chest CT examinations, independent of gated or non-gated acquisition.^61^ The European Society of Cardiology guidelines highlight CAC scoring as a tool for risk management and opportunistic assessment via non-ECG-gated chest CT, recommending a 4-category plaque burden score, though evidence for further imaging in asymptomatic individuals is lacking.^62^ The Dutch Ministry of Health, Welfare, and Sport permitted the reporting of CAC scores exceeding 300 Agatston units in the 4ITLR LCS trial, along with any other acute incidental findings deemed crucial for the participant’s prognosis. The ACR Incidental Findings Committee specifically recommends including CAC within diagnostic reports of both gated and non-gated CT scans, either with the quantitative Agatston score or qualitative visual assessment, with severe CAC prompting further evaluation.^27^ Despite these recommendations, CAC score remains often unreported on non-gated chest CTs, because many radiologists are unaware of the correlation between CAC scores from non-gated CT and the traditional ECG-triggered CT method, if the CT system has adequate temporal resolution.^63^ Other factors contributing to the underreporting of CAC include the absence of standardized guidelines for CAC reporting and the lack of routine practice among radiologists. This may stem from personal reporting styles, limited training, or a tendency to prioritize brevity in reports. Kirsch et al^63^ demonstrated that radiologists who use structured reporting templates report CAC in 100% of cases, highlighting not only the value of standardization but also the potential for integrating automated AI-derived CAC scores directly into the report. In clinical practice, radiologists are typically expected to report CAC only if categorized as moderate or severe, without requiring CAC scores of lower-risk categories.^59^^,^^60^ Furthermore, while documenting CAC at baseline scans in LCS is encouraged, there are no clear guidelines for assessment of CAC progression in subsequent annual/biennial screening rounds. Apart from identifying and quantifying the presence of CAC, the guidelines emphasize the importance of simple recommendations regarding further management.^59^^,^^61^ This overall lack of standardization has led to inconsistent inclusion and management of CAC scores, raising concerns about missed opportunities for preventive care.^40^ Standardized CAC reporting within LCS, regardless of the risk category, can improve patient care by facilitating timely preventive interventions, such as initiating statins, while reducing unnecessary medication use in low-risk individuals, providing a shift towards more personalized preventive care.^36^^,^^64^

## Advancements in CT technology

**Figure 1.**
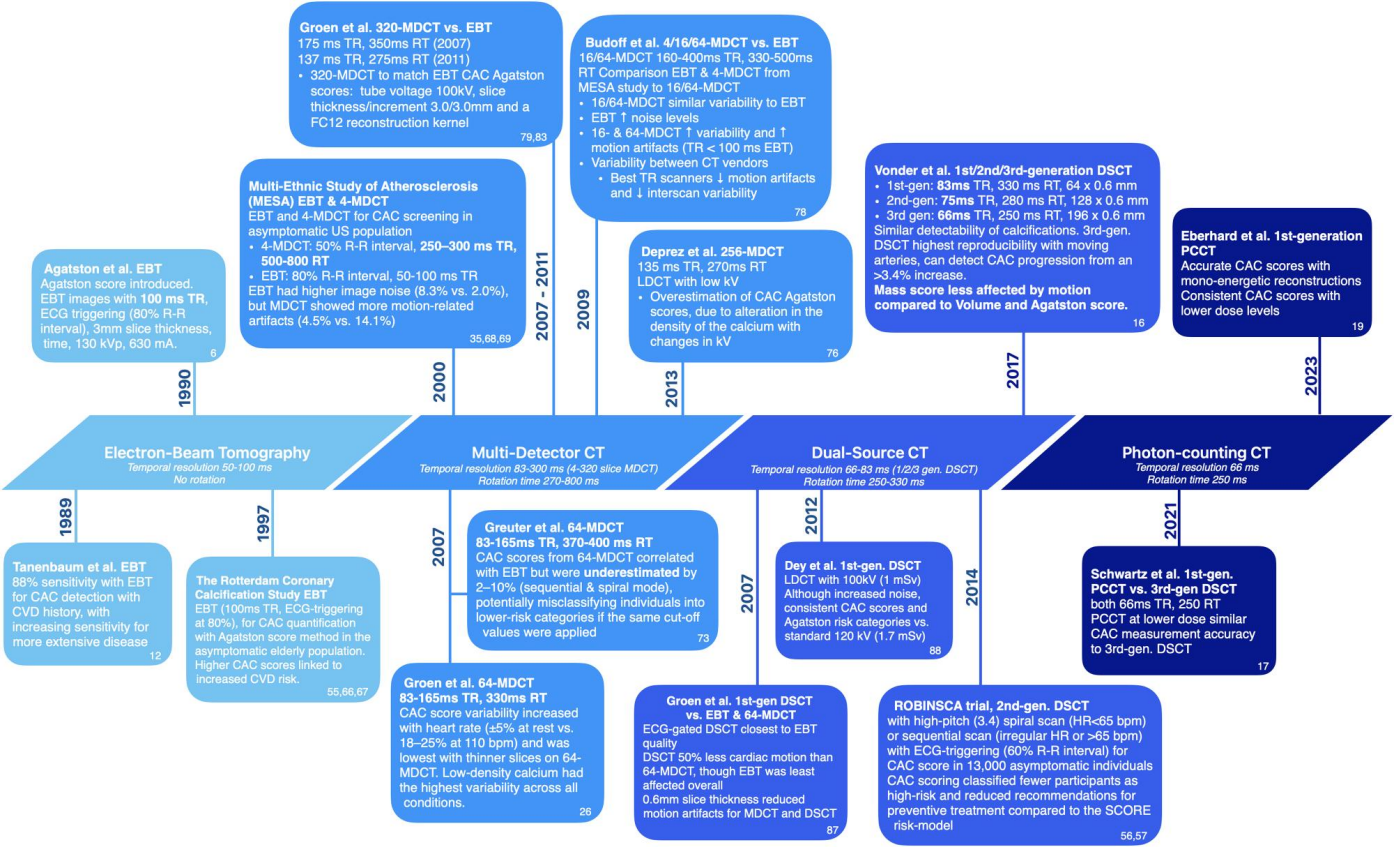
Imaging technology for coronary artery calcium (CAC) scoring has evolved from electron-beam tomography (EBT) to multidetector computed tomography (MDCT), dual-source CT (DSCT), and photon-counting CT. This image presents relevant studies that assess the performance of these imaging techniques over time, including comparisons through phantom studies and large population studies. Additionally, the temporal resolution (TR) and rotation times (RT) of the systems are reported, providing insight into their impact on CAC measurement accuracy and reproducibility. The reference number of the reported studies can be found in the bottom right corner of every box. Abbreviations: bpm = beats per minute; CVD = cardiovascular disease; DSCT = dual-source computed tomography; ECG = electrocardiographic; EBT = electron-beam tomography; gen = generation; MDCT = multidetector computed tomography; ms = millisecond; PCCT = photon-counting computed tomography; ROBINSCA = Risk Or Benefit IN Screening for Cardiovascular Disease; RT = rotation time; TR = temporal resolution.

Accurate CAC measurement on LDCT is essential for combined lung cancer and cardiovascular screening. However, image quality depends on patient factors, such as motion, body size, and breath-holding ability, as well as CT scanner parameters, including temporal resolution, cardiac gating, and detector technology, all of which affect CAC measurement reliability. Over time, CT imaging techniques for CAC assessment have evolved from EBT and single-source MDCT to advanced systems such as DSCT and photon-counting CT. These innovations have paved the way for the (potential) implementation of cardiovascular screening within LCS programs. See Figure 1 for a detailed timeline of imaging techniques and associated study results.

### Electron beam tomography

Originally, coronary calcium was detected using EBT, since the 100 millisecond (ms) temporal resolution and 1.5 mm spatial resolution made it perfect for imaging the moving heart. The first studies successfully measured CAC with EBT in 1990, and the Agatston score was established, the standard CAC quantification method to this day.^6^^,^^12^^,^^65^ Later the Rotterdam Coronary Calcification Study^55^^,^^66^^,^^67^ showed a clear link between increasing CAC scores and higher CVD risk, highlighting CAC as a strong, independent predictor of CVD in the elderly and a valuable tool for guiding preventive strategies.^55^ This study played a key role in anchoring EBT as a leading imaging tool for CAC evaluation, establishing its importance in cardiovascular risk assessment.^55^^,^^67^

### Multidetector CT

Advancements in CT imaging systems from EBT and single-detector CT to MDCT significantly improved temporal and spatial resolution and, combined with ECG triggering, challenged EBT as the standard for CAC imaging. Consequently, a large population study in the United States assessed subclinical CVD in asymptomatic individuals using both EBT and 4-slice MDCT scanners for CAC measurements.^68^ EBT showed more image noise but fewer motion artifacts than MDCT, with both showing similar reproducibility and 96% agreement in calcium detection.^69^^,^^70^ At that time, however, further evidence was needed to confirm the predictive value of CAC for cardiovascular events before recommending screening for part of the (asymptomatic) population.^10^^,^^69^

MDCT systems offered greater versatility and availability than EBT scanners, which were specifically designed for cardiac imaging. Unlike EBT’s single-slice acquisition, MDCT captures multiple slices in 1 rotation, covering a larger area of the heart. However, achieving rapid temporal resolution is crucial for minimizing coronary motion artifacts. EBT had superior temporal resolution (50-100 ms) due to its rotating electron beam, while MDCT was limited by the gantry rotation speed.^10^ Nevertheless, the number of detector rows increased over time and the rotation time became faster. The 320-MDCT, with the largest 160 mm z-axis detector coverage, enabled imaging of the entire heart in a single gantry rotation within a fraction of a second. This capability allowed for substantial dose reduction and minimized stair-step artifacts, which significantly improved early cardiac imaging.^71^ Furthermore, data from the diastolic rest period and preceding motion phase were combined through prospective ECG-triggering, overcoming previous limitations and enabling accurate coronary artery imaging.^72^ Additionally, with MDCT, tube current can be adjusted to compensate for patient size, addressing the decreased signal-to-noise ratio caused by X-ray attenuation in larger patients, a significant advantage over EBT, as reduced signal-to-noise ratio hampers plaque identification and increases false-positive CAC scores.^10^

Further proof of 16-, 64-, 256-, and 320-MDCT performance in cardiovascular imaging was investigated through several (phantom) studies, before slowly replacing EBT in clinical practice.^26^^,^^73–77^ EBT scanners tended to have higher noise levels, while some 16- and 64-MDCT scanners exhibited greater variability and more motion artifacts, due to lower temporal resolution. Furthermore, significant variability was observed between MDCT vendors, even among systems with the same number of detector rows.^78^ The 64-MDCT showed an underestimation of CAC scores by 2%-10%, while low kV settings (simulating LDCT) with 256-MDCT overestimated CAC scores due to alterations in calcium density.^73^^,^^76^ Despite these limitations early 64-, 256-, and 320-MDCT scanners gradually replaced EBT because of their lower noise, improved spatial resolution, and broader clinical utility.^26^^,^^73–76^^,^^79^ The ACR 2016 practice parameter for performing cardiac CT states that a scanner must “meet or exceed a 64-detector scanner” to achieve diagnostic quality imaging, given the challenges of capturing a moving object.^60^ However, the lack of standardization in cardiac CT protocols and CT systems results in a 10-fold variation in radiation doses, limiting the use of MDCT for routine CAC screening.^80^ Furthermore, the geometric inefficiency inherent to wide detector systems results in increased cone beam-related artifacts, such as overranging and scatter.^81^ The limited temporal resolution also impairs the integration of cardiovascular risk assessment into non-gated, low-dose LCS.^76^^,^^82^^,^^83^ Although CAC can be identified on MDCT performed for LCS, and a CAC score of 0 is associated with low CVD risk, dedicated cardiac CT remains necessary for accurate risk assessment.

### Dual-source CT

The evolution of DSCT has significantly advanced cardiac imaging capabilities over the years. In a single-source CT scanner, the X-ray source and detector must collect data across a 180° rotation to capture a cardiac image. By contrast, the 2 source-detector pairs of DSCT can collect the data to reconstruct an image in only a 90° rotation, effectively doubling the speed of data acquisition for diagnostic cardiac images. For single-source CT, the pitch is limited to 1.5 to avoid sampling gaps and ensure high image quality, while in DSCT the second detector system can fill these gaps, allowing the pitch to increase up to 3.2 without compromising image quality. With this high-pitch acquisition the entire heart can be captured in a single diastolic phase at heart rates up to approximately 65-80 beats per minute.^84^^,^^85^ Moreover, the temporal resolution of DSCT is independent of the patient’s heart rate, as images can be reconstructed from data acquired during a single cardiac cycle. Single-source MDCT systems can approach similar temporal resolution only with multisegment reconstruction, combining data from several cardiac cycles into 1 image, although it strongly depends on the relation of heart rate and gantry rotation time.^86^

The first-generation DSCT, introduced in 2006, used 2 detectors to simultaneously acquire 64 overlapping slices of 0.6 mm thickness with a 330 ms gantry rotation time, achieving a temporal resolution of 83 ms. Compared to MDCT systems, DSCT offered higher reliability, reduced impact of cardiac motion, and greater reproducibility.^87^^,^^88^ The 2 detectors of the second generation DSCT (2009) simultaneously acquired 128 overlapping 0.6 mm slices, with a faster 280 ms gantry rotation time, improving temporal resolution to 75 ms. The large-scale ROBINSCA-trial, demonstrated the potential of second-generation DSCT in preventive cardiology with a high-pitch spiral CT for CAC measurements in asymptomatic individuals.^56^^,^^57^^,^^89^ The third-generation DSCT (2014) provided simultaneous acquisition of 196 overlapping 0.6 mm slices per detector, with a 250 ms gantry rotation and a temporal resolution of 66 ms, enabling even greater reproducibility of CAC measurements under cardiac motion conditions.^16^^,^^86^ The DSCT systems have demonstrated improved accuracy in CAC measurements, and driven by the extra source-detector pair and increased number of detector rows, the temporal resolution improved significantly, enabling a high-pitch spiral CT acquisition protocol for accurate CAC measurements within a LCS program.

### Photon-counting CT

Compared to previous CT systems that use energy-integrating detectors, photon-counting CT represents a fundamentally different approach to CT imaging. Photon-counting detectors count the number of incoming photons, with the electronic signal being proportional to the energy deposited by each photon. This method offers several potential advantages: a higher contrast-to-noise ratio for materials with a high atomic number, such as calcium, improved spatial resolution, and a reduction in calcium blooming.^90^ The accuracy of CAC scoring using a first-generation photon-counting CT scanner has been demonstrated, and compared to third-generation dual-source CT revealed comparable calcium quantification and image quality, with photon-counting CT offering reduced radiation exposure.^17^^,^^19^ While not yet widely available, photon-counting CT holds promise for the future of cardiac imaging and CAC quantification.

### Non-gated CT acquisition

The reference standard for calcium scoring is a CT protocol with prospective ECG-triggering, which captures images during a specific phase of the cardiac cycle, or retrospective ECG-gated acquisition, which captures images throughout the cycle but reconstructs only the phases of interest (eg, diastole). Recent advancements in CT technology, however, have enabled dedicated CAC imaging without requiring ECG-triggering or gating.^63^

Budoff et al^91^ performed a non-gated (helical mode, pitch 1.375) chest LDCT and an ECG-gated (75% of R-R interval) cardiac CT, both with a 64-MDCT system, for CAC assessment in 50 patients. Correlation between CAC scores obtained by non-gated and gated protocols was excellent (ICC = 0.96), although the median variability was 44% between the 2 methods and increased with higher CAC scores. Multiple studies on non-gated chest CT versus cardiac CT found a high correlation between gated and non-gated acquisition protocols, although consistently a high variability (40%-44%) and a shift to lower risk categories for the non-gated acquisition protocol was reported.^92–94^ A systematic review on the correlation of non-gated and ECG-gated MDCT concluded CAC can be identified on non-gated chest CT with similar diagnostic accuracy compared to ECG-gated CT, although non-gated CT yielded false-negative CAC score in 8.8% of individuals and underestimated high CAC score in 19.1% of individuals.^25^ Jacobs et al^95^ compared low-dose, non-gated MDCT scans performed within 4 months of each other in a LCS trial and found good interscan agreement in risk stratification by Agatston score categories. However, high mean interscan variability limited the use of non-gated MDCT for monitoring CAC scores in individual patients. With the availability of more detector rows, Chen et al^96^ utilized a non-gated 256-MDCT with a fast helical scanning mode, achieving a sensitivity of 94.8% and a specificity of 100% for detecting positive CAC. The false-negative rate dropped to 5.2%, down from 8.8% with the older MDCT systems. The study also demonstrated a 95.1% agreement in risk category compared to dedicated ECG-gated cardiac CT; however, no information on variability and reproducibility was reported.

Hutt et al^97^ assessed the reliability of non-gated, high-pitch, second-generation DSCT as an imaging modality for CAC screening in routine chest CT examinations. A total of 185 smokers underwent 2 DSCT scans: a non-gated, high-pitch (3.0), high-temporal resolution (75 ms) scan covering the entire thorax (chest CT); and an ECG-triggered scan (75% of the R-R interval) focused on the cardiac cavities (cardiac CT). Results demonstrated that non-gated chest CT achieved a sensitivity of 96.4% and a specificity of 100% for detecting CAC. The relative variability between chest and cardiac CT was minimal at 1.81% and the intertechnique agreement for risk categorization was excellent (weighted kappa = 0.95; 95% CI, 0.93-0.98).^97^ Importantly, the results with DSCT compare favourably with the high intertechnique and interscan variability and risk-recategorization reported with single-source MDCT.^91–96^

**Figure 2.**
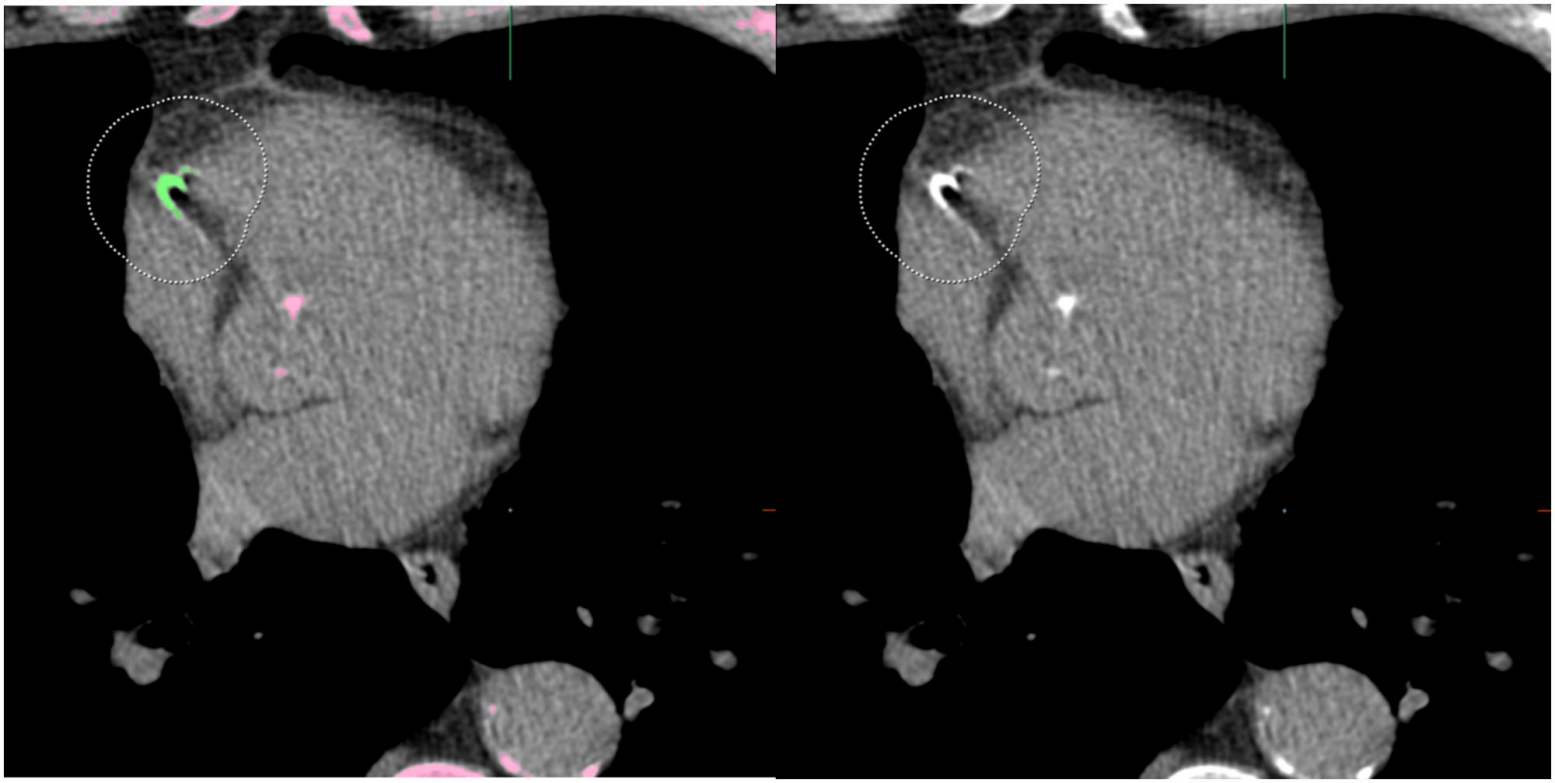
Example of an acquisition error of the coronaries with a 1-run, non-gated, low-dose chest CT part of a lung cancer screening program. The automated artificial intelligence (AI)-based coronary artery calcium (CAC) quantification software segmented a calcification in the right coronary artery (RCA) (segmentation in green), which has a motion artifact making the calcification seem larger, consequently overestimating the calcium.

Xia et al^18^ evaluated the effectiveness of non-gated chest CT compared to ECG-gated cardiac CT for CAC detection and risk stratification using high-pitch third-generation DSCT. The study involved 1000 participants from the ImaLife study, with 50% presenting coronary calcification on cardiac CT. Chest CT demonstrated a sensitivity of 96% and specificity of 99% for CAC detection. The median CAC score was lower on chest CT compared to cardiac CT (30 vs. 40, *P* ˂ .001), but agreement in risk categories remained high. Reclassification occurred in 6.5% of cases, mostly downward, with higher body mass index (≥30) linked to increased reclassification.^18^ The ongoing European LCS trial 4ITLR utilizes a 1-run, non-gated, LDCT acquisition protocol with a third-generation DSCT system for simultaneous lung nodule measurements and automated CAC quantification. Additionally, emphysema data is collected as well, using the same single LDCT scan, currently for research purposes only.^98^ Initial results demonstrate high reproducibility of the artificial intelligence (AI)-based CAC scoring between baseline and the 3-month follow-up, with 92.6% of cases maintaining the same risk category and only 1.4% experiencing a downgrade from high to indeterminate risk. These findings highlight the reliability of consistent CAC quantification over time using a 1-run, non-gated DSCT acquisition, enabling reproducible cardiovascular assessment.^99^ However, motion artifacts persist in some cases, as illustrated in Figure 2. The right coronary artery is most susceptible to motion artifacts, whereas the left main trunk and left anterior descending artery are less affected by motion and are associated with a higher risk of mortality when calcified.^100^^,^^101^ Therefore, rather than relying solely on the overall CAC score, it may be beneficial to consider the anatomical location of calcifications, particularly in scans acquired with limited temporal resolution where motion artifacts are more pronounced.^102^

International guidelines recommend reporting CAC scores on chest CTs across all CT systems, as they serve as a valuable marker of CVD risk.^36^^,^^61^ However, the current evidence does not support consistently high diagnostic imaging quality across all CT systems using non-gated protocols for combined lung and cardiac screening. Only high-pitch, high-temporal resolution second- and third-generation DSCT and photon-counting CT systems have demonstrated reliable performance in this context. This underscores the importance of recognizing the limitations of various CT systems, including variability at higher heart rates and the potential to underestimate cardiovascular risk with single-source MDCT systems.^18^

## Role of AI in combined screening

Despite the proven benefits of LCS for high-risk individuals, the increased workload radiologists will face of wide-spread implementation remains a major concern. To address this, many radiologists rely on computer-assisted detection/diagnosis (CAD) software for tasks such as lung nodule segmentation and classification. Advanced CAD systems increasingly incorporate AI to automate nodule detection, thereby reducing manual effort. CAC scoring is a repetitive, labour-intensive task for radiologists and would substantially add to their workload if incorporated into LCS. Similarly to lung nodule detection, AI-based software is being developed for semiautomatic and automatic CAC quantification, generating Agatston, volume, or mass scores from dedicated cardiac reconstructions. Semiautomatic tools assist in CAC quantification but remain time-consuming and user dependent.^103^ Fully automated AI-CAC tools can be integrated with LCS programs, without adding to the overall workload.

**Figure 3.**
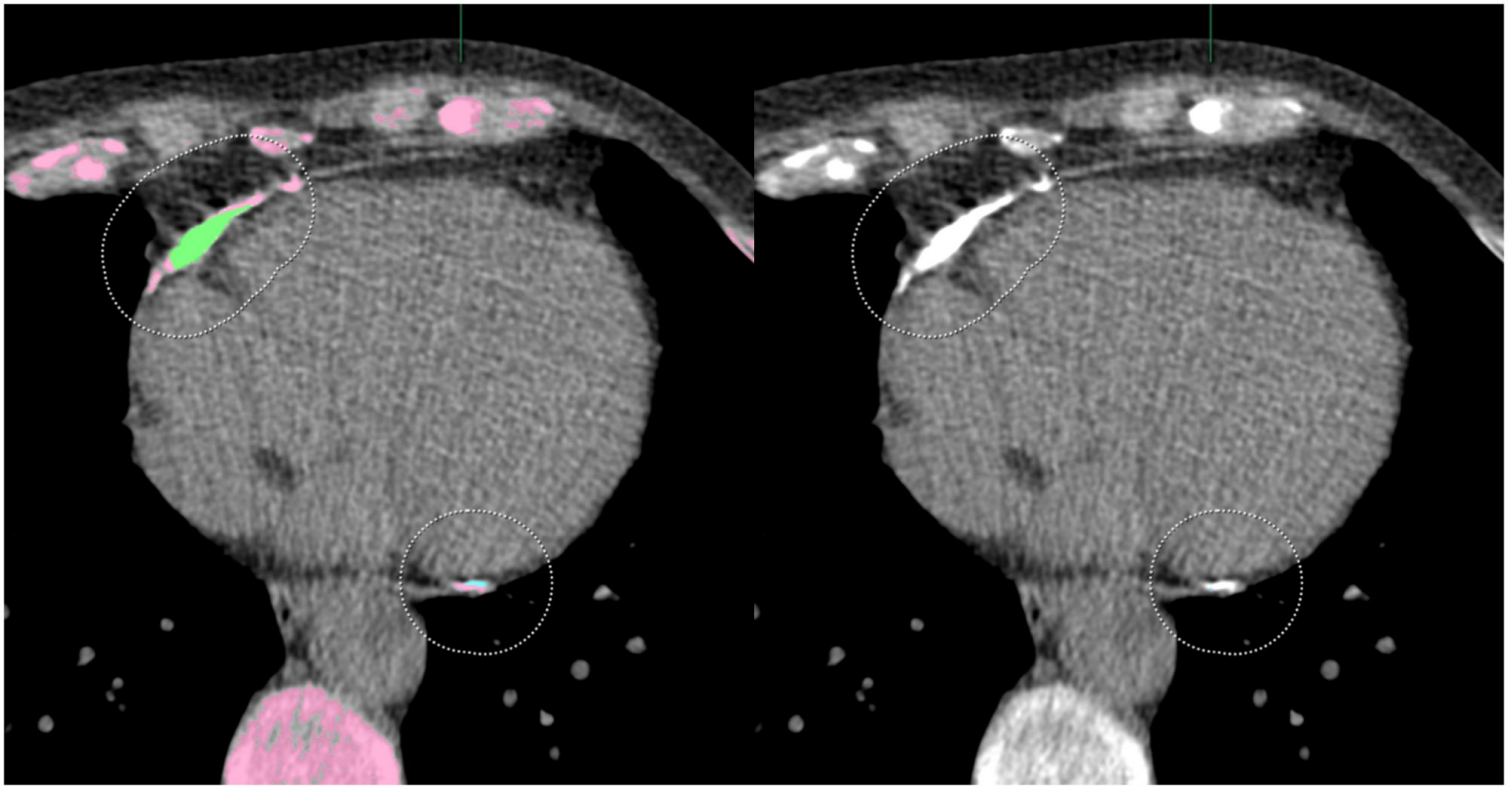
Example of an identification error of an automated artificial intelligence (AI)-based coronary artery calcium (CAC) quantification software. Pericardial calcification misidentified by the software as a calcified right coronary artery (RCA) (segmentation in green).

In LCS non-contrast, non-gated, low-dose chest CT is typically used, limiting the coronary artery visualization and segmentation. Non-coronary calcifications, such as those in the aortic valve or pericardium, may be misidentified as CAC on LDCT (see Figure 3).^82^^,^^104^ Many AI systems mitigate this by segmenting cardiac structures before localizing calcifications, and research increasingly focuses on automated AI-CAC quantification for non-contrast, non-gated LDCT.

Multiple studies comparing AI-CAC tools to conventional manual or semi-automated CAC scoring methods report excellent correlation and agreement when applied to non-contrast, non-gated LDCT.^105–110^ However, Sandstedt et al^105^ mentioned that their AI-CAC software had a tendency to overestimate the calcium score, while Yu et al^109^ mentioned an underestimation. Suh et al^107^ showed good reliability and risk categorization using an AI-CAC tool on chest LDCTs versus ECG-gated cardiac CTs, but specifically mentioned the heterogeneous variability among institutions. Interestingly, Cano-Espinosa et al^106^ developed an AI-CAC software without the need for CAC segmentation, only offering the non-contrast, non-gated CT as input and automatically providing the Agatston score as output. Vonder et al^111^ demonstrated a high level of agreement between automated deep learning-based and manual CAC scoring in a population-based screening setting (ROBINSCA) involving asymptomatic participants. Moreover, the AI software achieved a false-negative rate of 0.7%, a false-positive rate of 0.1%, and a diagnostic accuracy of 99.2% for guiding the initiation of preventive treatment. Within the European LCS trial 4ITLR, the AI-CAC software demonstrated high reproducibility, with an intraclass correlation of 0.96 (95% CI, 0.96-0.97) when comparing baseline and 3-month follow-up CAC scores acquired using non-contrast, non-gated, low-dose CT scans.^99^ For centres without high temporal resolution scanners, AI-based approaches can offer a practical alternative by enabling automated CAC quantification while also evaluating image quality to indicate result reliability.^112^ This can assist radiologists in determining when an AI-generated CAC score is trustworthy and when manual review or dedicated cardiac CT may still be warranted. Other studies linked automated AI-CAC scores to cardiovascular outcomes rather than direct score comparisons.^113–116^ De Vos et al^115^ showed that 5-year CVD mortality can be predicted in less than half a second, using only area-specific CAC-scores automatically derived from LCS LDCT. Similarly, Zeleznik et al^114^ showed an association between automated CAC scores and incident atherosclerotic-related death using LDCT from the NLST trial, while Peng et al^116^ linked CAC ≥100 on non-gated chest CTs with an increased risk of all-cause death and adverse cardiovascular outcomes. Sandhu et al^117^ further demonstrated that opportunistic CAC screening using AI-CAC software on non-gated chest CT scans significantly increased statin prescriptions.

Overall, these studies confirm that most automated AI-CAC software can accurately quantify CAC and effectively identify LCS participants at low- or high-risk for CVD, all without adding to the reading workload. However, the wide range of available software tools, each with varying performance and trained on different datasets, underscores the importance of using the software as intended and thoroughly validating its performance prior to clinical implementation.^82^

## Conclusion

Integrating cardiovascular screening into LCS programs with standardized CAC measurements can facilitate timely preventive interventions, such as initiating statin therapy for high-risk individuals, while minimizing unnecessary treatments for those at low risk. This requires accurate CAC quantification from the same non-contrast, non-gated LDCT scan used for LCS, without increasing the radiologist workload. Automated AI-CAC software accurately quantifies CAC and stratifies CVD risk, providing a scalable solution for integrating cardiovascular risk assessment into LCS programs, though careful validation remains essential due to performance variability. Integrating automated AI-derived CAC scores into structured reports will minimize CAC underreporting.

Advancements in CT technology, including increased detector rows, additional detector-source pairs, and new detector technology, have enabled sufficient image quality for effective opportunistic cardiovascular screening within LCS programs. However, the limited temporal resolution of MDCT, particularly with non-gated protocols, makes it challenging to match the accuracy and reproducibility once provided by EBT systems, risking CAC underestimation and incorrect risk classification. To ensure accurate CVD risk assessment, thorough validation of MDCT-based CAC quantification in LCS programs is essential, and CAC scores should be confirmed with dedicated cardiac CT protocols. Emerging technologies, such as DSCT and photon-counting CT systems, have shown excellent reproducibility of CAC scores and strong agreement in risk categorization. These techniques enable accurate CAC scoring from 1-run, non-gated LDCT scans, offering a promising approach for opportunistic cardiovascular screening within LCS programs.

Achieving accuracy and reproducibility in CAC measurements requires standardized acquisition protocols and comprehensive documentation of CT system specifications and vendor-specific parameters to support consistent, high-quality CAC reporting. Suboptimal CT quality can lead to CVD risk misclassification, while scan variability complicates longitudinal comparisons. Addressing these challenges is essential for effectively integrating CAC assessment into LCS programs, expanding CVD screening and optimizing preventive care.
